# Regulation of host immune cells and cytokine production induced by *Trichinella spiralis* infection

**DOI:** 10.1051/parasite/2019074

**Published:** 2019-12-19

**Authors:** Yining Song, Jing Xu, Xuelin Wang, Yong Yang, Xue Bai, Jianda Pang, Xinrui Wang, Mingchuan Yu, Mingyuan Liu, Xiaolei Liu, Shumin Sun

**Affiliations:** 1 College of Animal Science and Technology, Inner Mongolia University for Nationalities Inner Mongolia 028042 Tongliao PR China; 2 Key Laboratory of Zoonosis Research, Ministry of Education, Institute of Zoonosis/College of Veterinary Medicine, Jilin University 130000 Changchun PR China; 3 Jiangsu Co-Innovation Center for Prevention and Control of Important Animal Infectious Diseases and Zoonoses Yangzhou Jiangsu PR China

**Keywords:** *Trichinella spiralis*, Meso Scale Discovery, Cytokines, Immunoregulation

## Abstract

The nematode *Trichinella spiralis* can cause immunoregulation during the early phase of infection. However, previous studies are still insufficient for a full understanding of this phenomenon and its underlying mechanism. In this study, immune cells and cytokine profiles of *T. spiralis* infected mice were examined by Meso Scale Discovery (MSD) and flow cytometry. The MSD results of the spleen showed that Th1 immunity was inhibited from 6 h to 6 days post-infection (dpi) and the level of Th2 immune response was significantly increased at 6 dpi. The mesenteric lymph node showed a Th1/Th2 mixed immune response from 3 dpi to 6 dpi with a downtrend of Th1 at 6 dpi. Flow cytometry analysis showed that the proportion of Th1 cells of T cells was decreased significantly at 6 h after infection, the proportion of Th2 cells was markedly increased, indicating that Th1 immunity was significantly inhibited at 6 h after infection, and a hybrid immune response based on Th2 type was presented from 30 h to 6 dpi. The immunoregulation effects observed during this study have provided a better understanding of the development of the immune response induced by *Trichinella* infection.

## Abstract (fr)

Le nématode *Trichinella spiralis* peut provoquer une immunorégulation pendant la phase précoce de l’infection. Cependant, les études précédentes sont encore insuffisantes pour bien comprendre ce phénomène et son mécanisme sous-jacent. Dans cette étude, les profils de cellules immunitaires et de cytokines de souris infectées par *T. spiralis* ont été examinés par DMÉ (Découverte à Méso-Échelle) et cytométrie de flux. Les résultats DMÉ de la rate ont montré que l’immunité Th1 était inhibée 6 h à 6 jours après l’infection (jai) et que le niveau de réponse immunitaire Th2 était augmenté de manière significative à 6 jai. Le ganglion mésentérique présentait une réponse immunitaire mixte Th1/Th2 de 3 à 6 jai avec une tendance à la baisse de Th1 à 6 jai. L’analyse par cytométrie en flux a montré que la proportion de lymphocytes T Th1 était significativement réduite 6 h après l’infection, que la proportion de Th2 était nettement augmentée, ce qui indiquait que l’immunité Th1 était significativement inhibée 6 h après l’infection, et qu’une réponse immunitaire hybride sur le type Th2 était présentée de 30 h à 6 jai. Les effets d’immunorégulation observés au cours de cette étude ont permis de mieux comprendre le développement de la réponse immunitaire induite par l’infection à *Trichinella*.

## Introduction

Trichinellosis is a common zoonosis with a global distribution that has a great socioeconomic impact on human and animal health [12]. Although *Trichinella spiralis* is well controlled in Europe and the United States, it is still prevalent in developing and developed countries, including China, Argentina, and Eastern Europe [10]. After its successful isolation in 1835 [4], there has been a large number of studies on *T. spiralis* infection and parasitism [19]. It has been found that *T. spiralis* invasion is not just a simple case of mechanical penetration [23], and that immune suppression has been observed in the intestinal phase of mice [20, 21]. Continuous infection with *T. spiralis* can stimulate the body to produce acquired immune effects, but the host cannot completely inhibit the growth of *T. spiralis*, indicating that it can inhibit, and escape from, the host’s immune response [8]. This phenomenon further supports this study that the host’s immune mechanisms play an important role in the development, invasion, and parasitism of *T. spiralis* [26].

During the early stage of *T. spiralis* infection, cellular immunity is inhibited, while in the late stage, cellular immune function has recovered, and humoral immunity began to play a role in resisting *T. spiralis* infection [1]. During *T. spiralis* infection, Th1/Th2 cells play an important role in balancing immune system function, and once that balance is broken, the host will become infected [17]. Numerous studies have shown that *T. spiralis* infection affects its host’s immune system, inhibiting Th1/Th17 cell response and inducing Treg cells to reduce the inflammatory response [28]. When *T. spiralis* infects the host, different phase specific antigens are produced in different developmental stages of the parasite life cycle, which induce the host to produce a specific immune response [25, 27]. To account for this characteristic, we have carried out experiments to verify the four key stages of *T. spiralis* infection during the intestinal phase. By examining the expression and regulation of host immune cells and cytokines, our goal is to find a breakthrough point in the immune regulation of *T. spiralis* which could lead to new approaches to the treatment of trichinellosis.

## Materials and methods

### Ethics

All mice protocols were reviewed and approved by the Ethics Committee of Jilin University affiliated with the Provincial Animal Health Committee, Jilin Province, China (Ethical Clearance number IZ- 2009-08).

### Obtaining of muscle larvae and infection of BALB/c mice

*Trichinella spiralis* (ISS534) was provided by the Institute of Zoonosis of Jilin University. Female BALB/c mice, aged 6 weeks, were purchased from the Animal center of the medical department of Jilin University.

Muscle larvae were obtained from infected mice by artificial digestion performed using the latest version of the magnetic stirrer protocol, according to the OIE standard protocol. After a series of precipitation and washing steps, muscle larvae suitable for sample addition were finally obtained. The number of *T. spiralis* larvae obtained by digestion was counted under a microscope.

Forty-eight BALB/c mice were divided randomly into two groups, an infected group and a control group. Each mouse in the infected group was infected with 250 muscle larvae at the same time. Under aseptic conditions, samples of peripheral blood, spleens, and mesenteric lymph nodes of the infected group and the control group (six mice per group) were collected for MSD and flow cytometry analysis at 6 h, 30 h, 3 d, and 6 d after infection.

### Meso scale discovery (MSD) determination of cytokines

Based on the following advantages, MSD technology was selected for this experiment. Since the sensitivity of MSD is up to 0.05 pg/mL, it is more effective in finding a difference between the infected group and the control group. Only when the MSD plate is fed into the instrument and stimulated by electrodes can the signal be generated. The excitation time of each well is unified with the signal acquisition time, and the stability of data can be high.

The mesenteric lymph nodes and spleens were obtained at 6 h, 30 h, 3 dpi, and 6 dpi from the infected and control mice, under aseptic conditions, and stored at −80 °C prior to use in the detection of cytokines IL-2, IL-4, IL-10, IL-17A, IFN-γ, and TGF-β. For the cytokine determination test, 300 μL of linker was added to 200 μL of biomarker antibody (K15069L-1, Univ. Biotechnology, China) and incubated for 30 min at room temperature, then 200 μL of termination solution was added and the solution incubated at room temperature for 30 min. A volume of 50 μL of antibody was added to each well of the MSD plate and the plate was incubated for 1 h at room temperature. The MSD plate was rinsed three times with 1 × PBS (0.05% Tween-20), and 25 μL each of the diluent and sample were added. The plate was sealed and shaken at room temperature for 1 h. After washing the MSD plate three times again, 50 μL of the detection antibody was added to each well, and the plate shaken at room temperature for 1 h. After incubation, the MSD plate was rinsed three times with 150 μL of washing solution. The MSD plate was rinsed three times, 150 μL of the readout liquid was added. All data were analyzed with MSD software (QuickPlex SQ120, MSD, USA).

### Flow cytometry analysis of Th1, Th2, Th17, and Treg cells

An amount of 100 μL of peripheral blood was taken at 6 h, 30 h, 3 dpi, and 6 dpi from 12 mice from the infected and control mice, under aseptic conditions, and placed in an anticoagulant tube prior to further tests. Th1 cells (CD3^+^, CD4^+^, IFN-γ^+^), Th2 cells (CD3^+^, CD4^+^, IL-4^+^), Th17 cells (CD3^+^, CD4^+^, IL-17^+^), and Treg cells (CD4^+^, CD25^+^, Foxp3^+^) were analyzed and identified. The cellular subpopulations were detected using fluorescently labelled antibodies with samples incubated for 15 min. All the steps were carried out at 2–8 °C. The FITC anti-mouse CD4 antibody (No. 11-0042-81, Biolegend, USA) was used on all samples. Cy5 anti-mouse CD3 antibody (No. 15-0031-82, eBioscience, USA) and PE anti-mouse IFN-γ antibody (No. 12-7311-81, eBioscience, USA) to label Th1 cells; Cy5 anti-mouse CD3 antibody (No. 15-0031-82, eBioscience, USA) and APC anti-mouse IL-4 antibody (No. 17-7041-81, eBioscience, USA) to label Th2 cells; Cy5 anti-mouse CD3 antibody (No. 15-0031-82, eBioscience, USA) and PE anti-mouse IL-17 antibody (No. 12-7177-81, eBioscience, USA) to label Th17 cells; and APC anti-mouse CD25 antibody (No. 17-0251-81, eBioscience, USA) and PE anti-mouse Foxp3 antibody (No. 13-5773-82, eBioscience, USA) to label Treg cells. All data were analyzed using the CellQuest software on the BD FACSCalibur ﬂow cytometer (BD Biosciences, Heidelberg, Germany).

### Data processing and statistical analysis

Data differences were analyzed by ordinary one-way ANOVA with GraphPad Prism 8.1.2 software. *p* < 0.05 was considered to be statistically signiﬁcant. The replications of the experiments (MSD and flow cytometry) were performed three times.

## Results

### MSD detection of cytokine results

**Figure 1:**
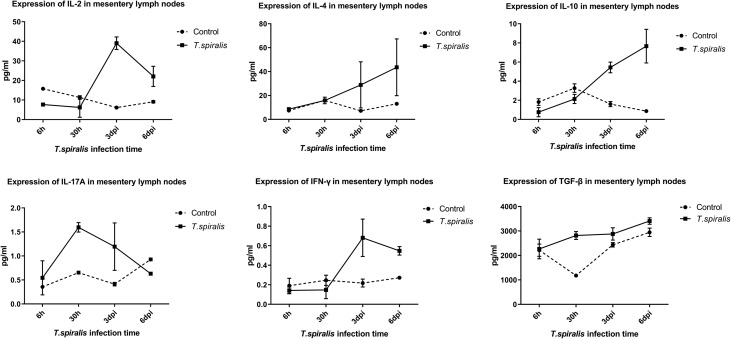
Cytokine expression levels of IL-2, IL-4, IL-10, IL-17A, IFN-γ, and TGF-β in the mesentery lymph nodes in mice infected with 250 muscle larvae of *Trichinella spiralis.*

When compared with the control group, the following observations were made in the mesenteric lymph nodes of infected mice. IL-2 levels significantly decreased at 6 h after infection, increased significantly at 3–6 dpi, and were not significantly different at 30 h; IL-4 levels were increased from 3 dpi to 6 dpi. IL-10 levels decreased (albeit not significantly) at 6 h and 30 h after infection, and increased significantly at 3–6 dpi, and IL-17A levels decreased significantly at 6 dpi, and significantly increased at 30 h and 3 dpi. IFN-γ levels were significantly increased at 3–6 dpi, and TGF-β levels were increased at 30 h after infection (Fig. 1).

**Figure 2:**
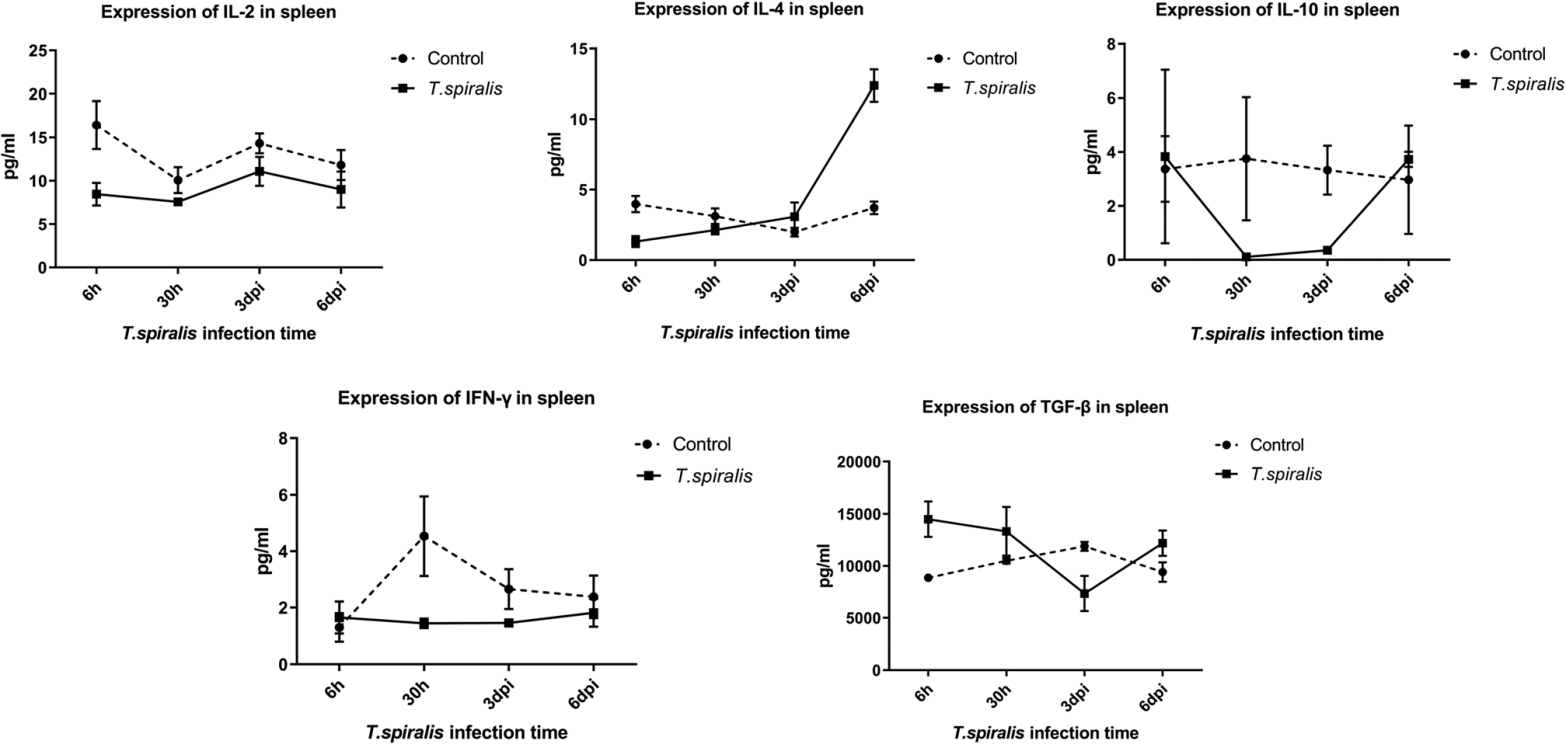
Cytokine expression levels of IL-2, IL-4, IL-10, IFN-γ, and TGF-β in the spleen in mice infected with 250 muscle larvae of *Trichinella spiralis.*

When compared with the control group, the following observations were made in the spleens of infected mice: levels of IL-2 were significantly decreased at 6 h, 30 h, and 3 d after infection, IL-4 levels were significantly decreased at 6 h after infection and increased at 6 dpi, and IL-10 levels were significantly decreased at 30 h and 3 d after infection. IFN-γ levels were decreased at 30 h after infection and TGF-β levels were decreased at 3 dpi, significantly increased at 6 h, and slightly increased at 6 dpi (Fig. 2).

### Flow cytometry analysis results

#### Evaluation of the percent change of T lymphocytes

**Figure 3:**
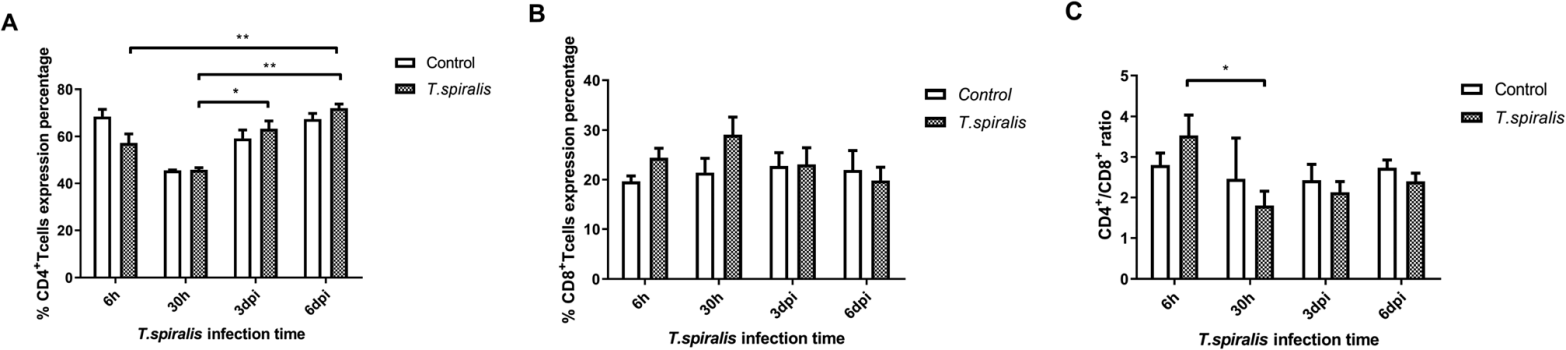
Percentage and expression levels of CD4+ and CD8+ T cells in mice infected with 250 muscle larvae of *Trichinella spiralis* compared to uninfected (control) mice from 6 h to 6 dpi. (A) Comparison of the mean percentage (±SD) of CD4^+^ T cells. (B) Comparison of the mean percentage (±SD) of CD8^+^ T cells. (C) Comparison of the expression (±SD) of CD4^+^/CD8^+^ T cells. *p < 0.05 and **p < 0.01 indicating a statistically significant difference.

The ratio of CD4^+^/CD8^+^ reflects the immune regulation status and immune level of the host. In infected mice at the intestinal stage of infection, levels of CD4^+^ T cells at 6 dpi were markedly increased, while CD8^+^ T cells at 6 dpi were decreased. According to the trends, CD4+ T levels were decreasing at 30 h after infection, while CD8+ T levels were on the rise (Fig. 3A and B). Compared with the ratio of CD4^+^/CD8^+^ at 6 h, it was significantly decreased at 30 h after infection (Fig. 3C).

#### Evaluation of the percent change of IFN-γ detection

**Figure 4:**
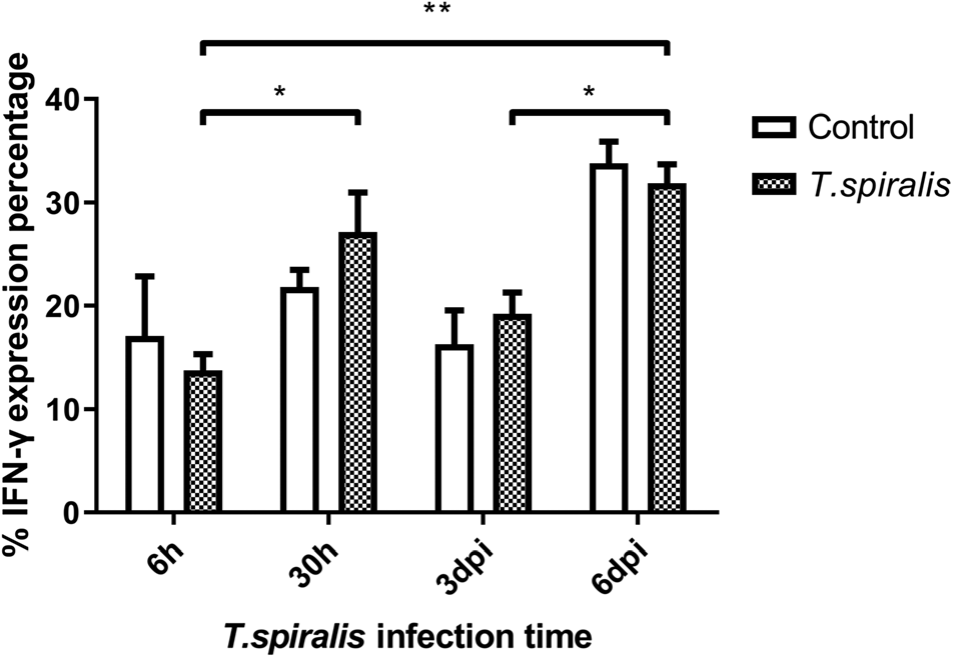
Percentage and expression levels of IFN-γ of Th1 cells in mice infected with 250 muscle larvae of *Trichinella spiralis* compared to uninfected (control) mice from 6 h to 6 dpi. *p < 0.05 and **p < 0.01 indicating a statistically significant difference.

As far as the overall trend is concerned, the expression level of IFN-γ was fluctuated. The secretion of IFN-γ by Th1 cells is involved in cellular immune regulation, mediates the immune response, and has an immunosuppressive effect on the worms invading the intestine of mice. In the infected group, the expression of IFN-γ at 6 h was significantly lower, while at 30 h, it was slightly higher (Fig. 4). Compared with the 6 h infection group, the expression of IFN-γ in the infected group was significantly increased at 6 dpi.

#### Evaluation of the percent change of IL-17

**Figure 5:**
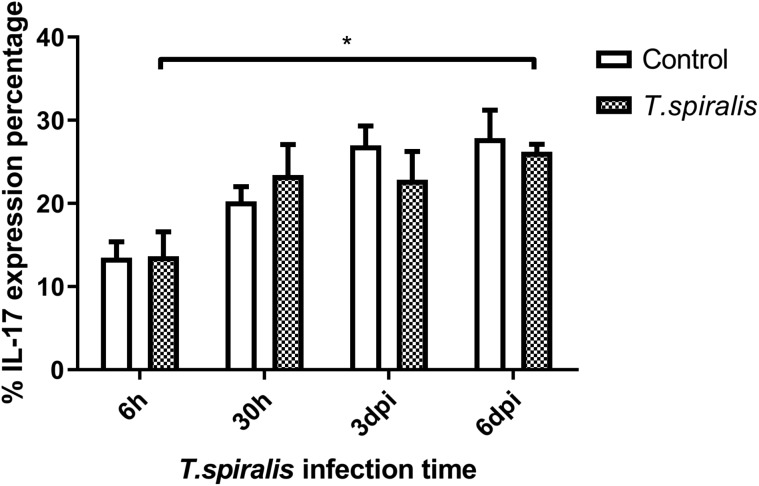
Percentage and expression of IL-17 of Th17 cells in mice infected with 250 muscle larvae of *Trichinella spiralis* compared to uninfected (control) mice from 6 h to 6 dpi. *p < 0.05 indicating a statistically significant difference.

IL-17 mediates the inflammatory response. Expression in the infected group was gradually elevated at the four time points of infection (Fig. 5). Compared with the 6 h infection group, the expression of IL-17 in the infected group was increased at 6 dpi.

#### Evaluation of the percent change of Foxp3^+^

**Figure 6:**
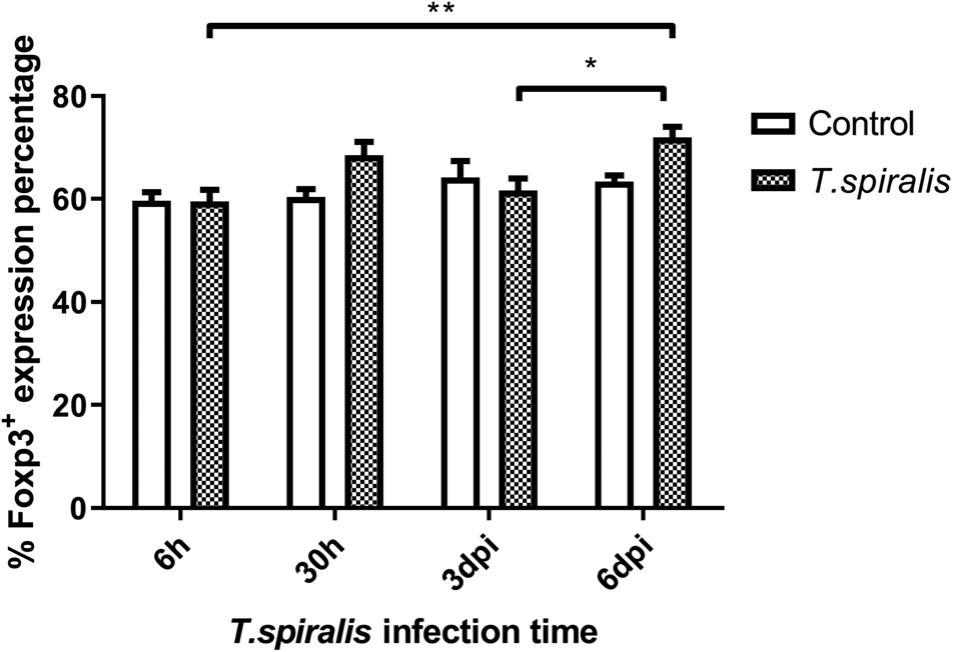
Percentage and expression levels of Foxp3^+^ of Treg cells in mice infected with 250 muscle larvae of *Trichinella spiralis* compared to uninfected (control) mice from 6 h to 6 dpi. *p < 0.05 and **p < 0.01 indicating a statistically significant difference.

Foxp3^+^ was expressed at a high level at each time point (Fig. 6). The infected group expression levels were only higher at 30 h and 6 dpi, and no significant differences were seen at the other two time points. Treg cell numbers were highest at 6 dpi.

#### Evaluation of the percent change of IL-4

**Figure 7:**
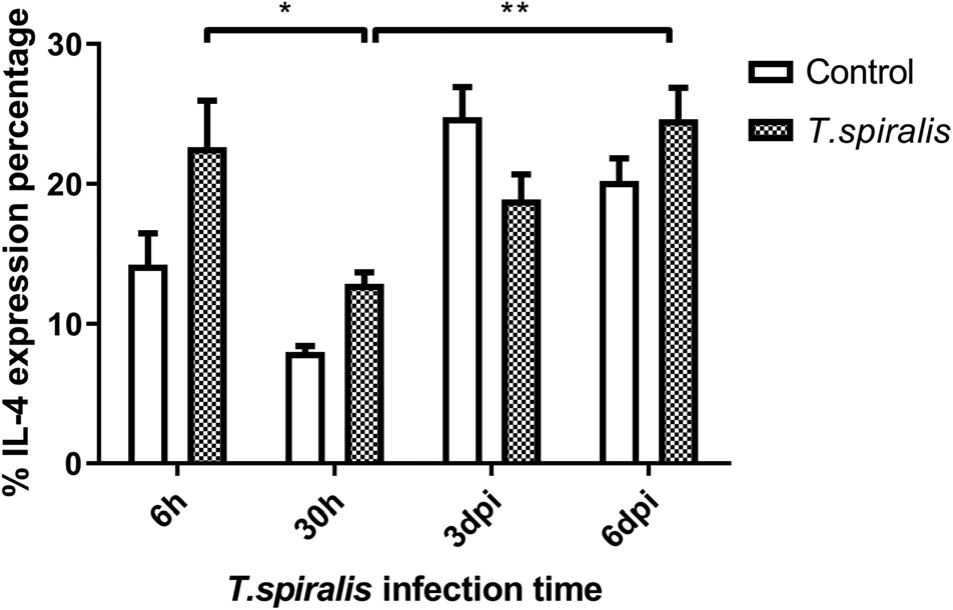
Percentage and expression of IL-4 of Th2 cells in mice infected with 250 muscle larvae of *Trichinella spiralis* compared to uninfected (control) mice from 6 h to 6 dpi. *p < 0.05 and **p < 0.01 indicating a statistically significant difference.

Compared with the 30 h infection group, the expression of IL-4 in the infected group was significantly increased at 6 dpi. With regards to the overall trend, levels of IL-4 expression fluctuated greatly at 6 h and 3 dpi (Fig. 7). IL-4 activates mast cells to release histamine and serotonin to increase intestinal peristalsis and causes diarrhea, thereby stimulating the discharge of parasites. Changes in IL-4 levels during intestinal invasion may be due to immune escape by *T. spiralis*.

## Discussion

*Trichinella spiralis* infection has an immunosuppressive effect on the host’s innate immune system [2, 24, 29]. At this stage, the worms develop rapidly, the morphology, and excretion change constantly, and the newborn larvae begin to be developed at 4 dpi. Ultimately, through complex developmental processes, the newborn larvae migrate rapidly with blood or lymph and invade the skeletal muscle. It is speculated that the immune regulation of the host at different time points up to 6 dpi is complex and volatile. Most studies have tested the immune regulation above 1 dpi. In this study, different views on this detection time were presented, and mice were used as experimental animals to detect the immune response of mesenteric lymph nodes and spleen during the four stages. The four stages were: the invasion period of muscle larvae into intestinal epithelial cells infected with *T. spiralis* (6 h), the initial stage of adult *T. spirali*s (30 h), the mating period of the adult males and females (3 dpi), and the growth period of newborn larvae (6 dpi).

The results of our MSD study showed the inflammatory reaction of the intestinal tract during *T. spiralis* infection from 6 h to 6 dpi. Expression of IL-2, important for inducing the Th1 response, was decreased significantly, the levels of TGF-β and IL-17A increased significantly at 30 h after infection. IL-17A is a pro-inflammatory cytokine that can strongly recruit neutrophils, suggesting that the host was in a state of pro-inflammatory reaction. The levels of Th1 cytokines (IL-2, IFN-γ^+^) and Th2 cytokines (IL-4, IL-10) were significantly increased at both 3 dpi and 6 dpi, suggesting that the host is in an inflammatory response stage, as shown by a mixed Th1/Th2 immune response. This is similar to the findings of Ilic et al. [16]. It was found that Th1 inflammation could effectively clear the intracellular infection. *Trichinella spiralis* had invaded mesenteric epithelial cells within 6 h after infection and had molted four times and developed into adults at 30 h after infection. Therefore, the inflammatory reaction at 30 h after infection could not completely eliminate all adult worms, which is in accordance with the characteristics of *T. spiralis* [14]. During the larval stage of *T. spiralis* development, the host is in a Th2-type immune response for a long period of time as a result of the long-term stimulation of the host immune system during the different developmental stages of the worm. *Trichinella* infection stimulates the host organism causing immune imbalance which gradually leads to a Th2-type immune response [6, 22]. This response has helped find new treatments to diseases caused by Th1-type immune response imbalance, since *Trichinella* infection can regulate the immune balance of hosts with imbalances (e.g., patients with inflammatory bowel disease).

The MSD results of the spleen in the early stages of *T. spiralis* infection showed that the Th1 immune response was inhibited from 6 h after infection to 3 dpi, until it returned to normal levels at 6 dpi, whereas the immunosuppression of the Th2 response only occurred at 6 h after infection. In addition, the level of Th2 immune response induced by IL-4 was significantly increased in the spleen at 6 dpi, which was beneficial to the stimulation of the humoral immune response. In 1198, Lawrence et al. [17] have also found that intestinal lesions are related to IL-4 and the immune response mediated by Th2 cells, agreeing with our results. These results suggest that the excreta of *T. spiralis* play an immunosuppressive role in peripheral immune organs (mesenteric lymph nodes and spleen). Th1 cells have an important role in the resistance to intracellular pathogens (viruses, bacteria, and parasites) and in the case of intracellular infection, Th1 cells can effectively trigger a cellular immune-mediated host defense response. However, the muscle larvae are still able to invade the intestinal epithelial cells and develop into adult parasites. Inhibition of Th1-type immunity in this period will provide sufficient time for the invasion, development, and the production of larvae of *T. spiralis*. It is beneficial therefore, for *T. spirali*s to establish infection [14, 22], evade the host immune system, and to reduce the resistance of the host and obtain the chance of parasite parasitism [6]. The level of immunosuppressive TGF-β expression in the spleen was significantly increased at the 6 h after infection, indicating that it plays an important role in inducing Th1-type and Th2-type immunosuppression. This suggests that there must be some molecules in the excreta of *T. spiralis* that have a strong immunomodulatory effect that can stimulate the expression of TGF-β [13]. Isolation and identification of these molecules will be part of a future study.

The immune mechanism of parasitic diseases mainly involves the interaction of Th1 and Th2 reactions and related cytokines, which inhibits their reproduction and amplification [11]. Th1 cells mainly secrete IFN-γ to regulate cellular immunity and mediate cellular immune response. According to the results of this experiment, the expression of IFN-γ fluctuates at different infection times, which indicates that the content of IFN-γ secreted by Th1 cells changes, participates in the regulation of cellular immunity, mediates the immune response, and regulates cellular immunity, and invasiveness. *Trichinella spiralis* infecting the intestinal tract of mice has certain immunosuppressive effects. IL-4 produced by the Th2 subgroup mainly acts as an immune response against extracellular multicellular parasites. Mast cells are activated by IL-4 to release histamine and serotonin, which can increase intestinal peristalsis and diarrhea and stimulate the discharge of parasites. As a whole, the level of IL-4 fluctuates greatly at 30 h and 6 dpi, the changes may be caused by immune escape of *T. spiralis*. Th17 cells are one of the subgroup cells differentiated from CD4+ T cells, which specifically secrete cytokine IL-17. Even though Th17 cells complete differentiation, they will be limited by many complex factors. The differentiation of Th17 cells was inhibited by the production of IFN-γ and IL-4, which were secreted by Th1 and Th2 cells [7]. In this experiment, the level of IL-17 in the infected group increased at 6 dpi compared with 6 h, indicating that Th17 secreted IL-17 to control infection. The number of Th17 and Treg cells would remain at a stable level to maintain the stability of the immune system. *Trichinella spiralis* most likely down-regulates host immune response through signaling to increase the number of Treg cells [16]. Many studies have shown that both the rapid and slow stages of *Trichinella* infection can increase the number of Treg cells. The experimental results support this statement, Foxp3^+^ was expressed at a high level at each time point. The main effect of Foxp3^+^ on host immune suppression is to maintain immune tolerance and control excessive inflammatory reactions [5, 15]. In this study, we found that the mesenteric lymph nodes in mice after *Trichinella* infection showed a significant Th1/Th2 mixed immune response from 3 dpi, and before this time, *T. spiralis* had already established infection and developed into adulthood. The inflammatory and immune responses after 3 dpi helped to reduce the damage caused by parasites and establish long-term parasitism. The level of Th2 response in the spleen from 6 dpi was also significantly increased, which was consistent with published studies on *T. spiralis* immunology which show that infection induces a Th2-type-dominated immune response in the host [3, 18]. This reflects the complexity of host immune regulation in the early stages of *Trichinella* infection. *Trichinella* has achieved long-term parasitism in the host by inducing a Th2-type immune response produced by the body [9]. The results of this study will help to elucidate the immunosuppressive mechanism induced by *T. spiralis* in early induction of the host, and provide a firm foundation for the study of related diseases.
